# Data integrity in Prosthodontics: A systematic review

**DOI:** 10.6026/973206300220593

**Published:** 2026-01-31

**Authors:** Dheeraj Deepak Kalra, Snehal Vilas Thamke, Purvi M Bhate, Kushal Gajendra Shinde, Pallavi Divekar, Kirti Rajkumar Andhalkar

**Affiliations:** 1 Department of Public Health Dentistry, Government Dental College & Hospital, Nagpur, Maharashtra, India; 2 Department of Public Health Dentistry, SMBT Institute of Dental Science and Research, Dhamangaon, Igatpuri, Nashik, Maharashtra, India; 3 Department of Public Health Dentistry, JMFs ACPM Dental College & Hospital, Dhule, Maharashtra, India; 4 Department of Public Health Dentistry, CSMSS Dental College, CHH. Sambhajinagar, Maharashtra, India; 5 Department of Public Health Dentistry, Late Shree YashwantraoChavan Dental college and Hospital Ahilyanagar, Maharashtra, India

**Keywords:** Prosthodontics, statistical reporting, peer review, methodological rigor, research audit

## Abstract

The validity of clinical research depends on robust statistical methodology, yet peer review often fails to identify analytical flaws, particularly in Prosthodontics where statistical errors can mislead evidence-based practice. Therefore, it is of interest to audits the statistical rigor of available data on this issue. Hence, 328 publications were systematically analysed. Our analysis shows that about one-third demonstrated rigorous, assumption-validated analyses, while nearly half showed ambiguous or inappropriate usage. Common issues included lack of assumption checks, inadequate adjustment for multiple comparisons, and missing sample size justifications. Thus, we show the urgent need for improved editorial standards, mandatory reporting checklists, and dedicated statistical review to ensure transparency and research integrity.

## Background:

The peer review process is often regarded as the cornerstone of scientific publishing, designed to uphold the quality and credibility of research. Yet, it has long been criticised for its inability to detect methodological and statistical flaws before publication. More recently, concern has also grown about the converse problem, reviewers identifying faults in statistical analyses where none actually exist. As highlighted by Bacchetti [1], spurious statistical critiques, often stemming from reviewers lacking formal training in statistics, can hinder the dissemination of sound research. This issue becomes especially significant in clinical disciplines, where flawed reviews not only delay publication but also misguide future studies and policy decisions. Recognizing these concerns, several journals have taken proactive measures to enhance statistical integrity. For instance, Obstetrics and Gynecology instituted mandatory statistical review for all submitted manuscripts after an internal audit in 1993 revealed widespread avoidable errors. A follow-up editorial revealed that 16% of submissions were subsequently rejected on the grounds of flawed study design or statistics, which prompted significant editorial reforms across the field [2]. Similarly, a review of articles in Infection and Immunity documented statistical or design flaws in 54% of papers assessed highlighting that problems are both frequent and systematic [3]. Parallel concerns have been observed in neuroscience publishing. A comprehensive audit of 580 articles in the Journal of Neurophysiology (2019-2020) revealed that 60% misused standard error reporting, 40% failed to define statistical thresholds, and 64% misinterpreted marginal p-values (0.05-0.1) as significant trends [4]. Moreover, even when statistical reporting guidelines were introduced, only about one-third of studies adhered to the required practices, and fewer than 10% complied with the recommended standards suggesting that editorial mandates alone may be insufficient to improve reporting quality. Despite growing awareness, the use of statistics in biomedical research continues to show substantial room for improvement. Numerous journal-based reviews have highlighted that a significant proportion, often more than half of published articles contain errors in statistical analysis, reporting, or both. Common issues include the omission of essential methodological details, misuse of statistical tests, failure to account for multiple comparisons, and inadequate justification for assumptions such as normality [3]. Therefore, it is of interest to describe the statistical and analytical practices and to assess the extent to which published studies adhere to established standards of statistical rigor.

## Materials and Methods:

A comprehensive review was conducted of all twelve issues of the Journal of Prosthetic Dentistry (JPD) published in the year 2023, comprising a total of 328 articles. To assess the appropriateness and accuracy of statistical methods used in the selected studies, a comprehensive quality assessment master sheet was developed. This sheet included forty-one [5, 6, 7, 8, 9, 10, 11, 12, 13, 14, 15, 16, 17, 18, 19, 20, 21, 22, 23, 24, 25, 26, 27, 28, 29, 30, 31, 32, 33, 34, 35, 36, 37, 38, 39, 40, 41, 42, 43-44] full-text articles retrieved from a pool of 101 eligible publications (excluding case reports and technique notes) across twelve issues published in 2023, from a total initial sample of 328 articles. The master sheet [Appendix I] was structured to identify and record various key statistical aspects relevant to study design, analysis, and reporting. Each article was evaluated for explicit mention and appropriate implementation of methodological elements such as randomization (including the technique or procedure), blinding (with specification of levels), and matching. The use of statistical tests was critically reviewed for appropriateness and justification, including whether post hoc tests were employed correctly and whether Levene's test was used prior to applying the t-test to assess homogeneity of variances. Misapplications such as the use of an unpaired t-test instead of a paired one were noted. In the present review, meticulous attention was given to whether studies addressed critical assumptions underlying statistical inference. Specifically, the usage of alternative robust tests such as the Welch ANOVA or Brown-Forsythe test in the presence of heteroscedasticity (variance inequality) was noted. The analysis also captured whether any normality assessments were conducted, emphasizing the application of the Shapiro-Wilk test, which is more suitable for smaller samples, over less sensitive options like the Kolmogorov-Smirnov test. The presence and correctness of sample size calculations were documented, particularly focusing on whether authors reported the use of validated formulas. Additionally, the clarity and precision of tabular data presentation were evaluated, as was the application of data transformation techniques in cases where assumptions of normal distribution were not met. For comparative and case-control designs, the review considered whether authors justified their case-to-control ratios, and whether tail directionality (one-tailed vs. two-tailed testing) was clearly justified and aligned with study hypotheses. Special attention was paid to baseline group imbalances and whether intergroup comparisons were made despite such differences without appropriate statistical control. The evaluation also recorded the use of chi-square test alternatives, such as Yates' continuity correction, McNemar's test, or Cochran's Q, particularly where expected frequencies were low or paired data were involved.

## Statistical errors were flagged in cases where:

[1] Multiple comparisons were conducted without appropriate adjustment procedures (*e.g.*, Bonferroni or Tukey correction),

[2] Inferential claims were made without appropriate tests, or tests used were inappropriate for the data type,

[3] Variability was reported using misleading or unlabeled error bars (*e.g.*, using standard error instead of standard deviation without clarification), or

[4] Statistical test descriptions were entirely absent, hindering interpretation or replication.

This structured appraisal, guided by a custom-developed statistical rigor master sheet, enabled consistent and transparent identification of both methodological strengths and recurrent statistical shortcomings across the selected literature.

## Results:

All forty-one included articles were systematically evaluated for the clarity, correctness, and appropriateness of their reported statistical methodologies using the predefined master sheet (Appendix I). Based on this structured audit, each study was classified into one of four categories reflecting the overall rigor of statistical application (Table 1). Across the reviewed literature, statistical approaches ranged from purely descriptive analyses to multivariable and time-to-event models. Commonly reported inferential techniques included t-tests, analysis of variance, non-parametric alternatives, regression models, and survival analyses. In exploratory, simulation-based, or finite element studies, restriction to descriptive statistics was generally appropriate. However, in several instances, descriptive findings were subsequently interpreted inferentially without formal hypothesis testing. Formal assessment of statistical assumptions was inconsistently reported. Although parametric tests were frequently employed, explicit testing for normality and homogeneity of variance was absent in a majority of studies. In some cases, non-parametric methods were selected for skewed or ordinal data; however, justification for test selection was not always clearly articulated. Advanced analytical methods, including mixed-effects models, generalized estimating equations, and Cox regression, were used in several clinical and longitudinal studies, though reporting of model diagnostics, covariate adjustment, or proportional hazards testing was inconsistent. Adjustment for multiple comparisons varied considerably across studies. While some investigations appropriately applied correction methods such as Bonferroni or Tukey procedures, many analyses involving multiple outcomes, regions, or subgroup comparisons did not report any form of error control, increasing the risk of inflated type I error. A notable finding was the frequent absence of sample size justification. Most studies did not report a priori power calculations or provide a rationale for group sizes, even when statistically significant results were presented. The quality of statistical reporting also varied widely. While some articles offered detailed descriptions of analytical procedures and variability measures, others relied on vague statements that limited reproducibility and critical appraisal. Reporting of effect sizes, confidence intervals, baseline group comparisons, and definitions of error bars was inconsistent across the reviewed literature.

## Discussion:

The present audit of forty-one full-text original studies published in the Journal of Prosthetic Dentistry during 2023 reveals substantial variation in the application, reporting, and justification of statistical methods. Despite growing awareness of the importance of statistical rigor in biomedical publishing, these findings reflect ongoing shortcomings in both analytical execution and reporting transparency within prosthodontic literature. The findings of this review align with concerns raised in prior literature regarding the variability and overall rigor of statistical reporting across dental publications. Vähänikkilä *et al.* [45] previously demonstrated that articles in prominent medical journals such as The Lancet and New England Journal of Medicine (NEJM) not only employed more complex statistical methodologies but also exhibited higher statistical intensity compared to those in dental journals. Conversely, dental literature was often characterized by smaller sample sizes and less frequent use of multivariable or computational techniques, with a greater prevalence of non-experimental study designs. Alarmingly, articles in dental journals were also more likely to report statistically significant outcomes, suggesting potential risks related to multiple testing and selective reporting, which are known factors to contribute to publication bias. In the present review of prosthodontic research published in a leading dental journal, these patterns were substantiated. Only approximately one-third of studies demonstrated rigorous and assumption-validated statistical approaches. Nearly half (48.8%) of the articles reviewed exhibited either ambiguous application or questionable robustness in their use of statistics. Furthermore, nearly 20% of studies contained inappropriate or seriously flawed analyses, raising significant concerns about the reliability and interpretability of their findings. The frequent absence of assumption testing, use of generic terms like "as appropriate," and lack of transparency in statistical methodology significantly hinder reproducibility and critical appraisal.

Our findings parallel those of prior audits in other domains of biomedical research, such as the Journal of Neurophysiology, where reporting inconsistencies persisted across several key metrics despite prior editorial interventions. Martin *et al.* [4] noted that 60% of reviewed articles misused standard errors, while 23% lacked definitions for variability measures. Alarmingly, 64% of studies misinterpreted borderline p-values as statistically meaningful. Even when enhanced reporting standards were introduced, adherence was limited, suggesting that passive strategies like policy statements and voluntary guidelines often fail to enact meaningful change. Supporting these concerns, Nieminen and Uribe [46] quantitatively assessed statistical reporting quality across different tiers of dental journals, revealing notably lower quality in so-called predatory journals, but also identifying important gaps even in reputable open-access and highly cited publications. Their findings reinforce the notion that deficiencies in statistical transparency often reflect broader lapses in research quality and editorial oversight. Collectively, these findings point to a systemic need for stricter statistical standards, improved peer review scrutiny, and clearer reporting guidelines in prosthodontic research. Addressing these gaps is crucial to ensure both the scientific validity and clinical applicability of published evidence. In the current review of prosthodontic literature, similarly weak adherence to assumption testing, justification of test selection, and proper post hoc treatment was observed. These patterns reinforce the broader concern that statistical rigor is often under prioritized in clinical research. As in neuroscience, improving reporting quality in prosthodontics may require not only updated author instructions but also active interventions-such as mandatory reporting checklists, structured statistical peer review, or increased emphasis on data transparency during editorial decision-making.

## Key takeaways (Figure 1):

**Figure 2:**
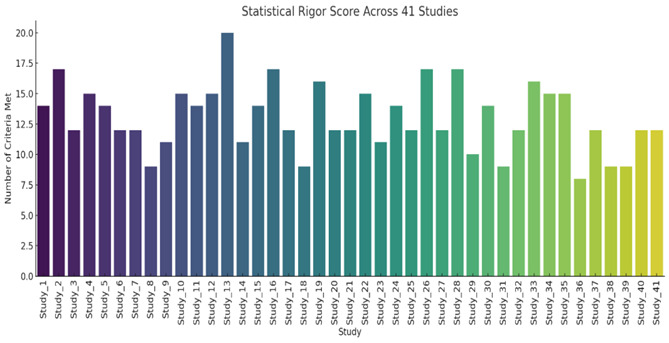
Heat map to show the statistical compliance across different domains of the selected studies

[1] The distribution of statistical rigor scores across 41 studies revealed considerable variability, with fewer than half of the studies meeting more than 70% of evaluation criteria (Figure 2), underscoring inconsistent adherence to methodological standards.

[2] Only 1 in 3 studies demonstrated assumption-verified, rigorous statistical workflows.

[3] Nearly 49% relied on assumed or questionable statistical appropriateness.

[4] Around 20% exhibited inappropriate or flawed statistical applications.

[5] 60% used parametric tests (*e.g.*, t-tests, ANOVA) without assumption checks.

[6] 75% failed to adjust for multiple comparisons, risking inflated false positives.

[7] 70% lacked any sample size justification, despite drawing significance conclusions.

[8] Only 14 studies (∼34%) applied Shapiro-Wilk for normality testing.

## Editorial practices and the role of peer review:

Recognizing these issues, several journals have already implemented stronger oversight mechanisms. Obstetrics & Gynecology, for instance, introduced mandatory statistical screening after finding 16% of submissions had serious errors. Similarly, Infection and Immunity found 54% of articles contained statistical mistakes. Despite such examples, prosthodontic journals have yet to systematically adopt similar safeguards. Peer review alone is not sufficient. Many errors occur, especially in assumption checking or multiple testing that require trained statistical reviewers. A structured checklist or a mandatory statistical review layer could significantly improve reporting quality.

## Recommendations for improvement:

## For authors:

[1] Clearly specify every statistical test and link it to the variable or comparison used.

[2] Always test and report assumptions prior to using parametric methods.

[3] Provide sample size or power justifications where relevant.

[4] Avoid ambiguous statements like "statistics were used where appropriate."

## For peer reviewers and editors:

[1] Require disclosure of assumption testing (*e.g.*, Shapiro-Wilk, Levene).

[2] Enforce the use of nonparametric tests when assumptions are violated.

[3] Demand corrections for multiple comparisons where multiple endpoints are analyzed.

[4] Encourage adherence to updated reporting standards (*e.g.*, SAMPL or CONSORT guidelines).

## For journals:

[1] Include a mandatory statistical checklist or add a statistical reviewer to the editorial process.

[2] Require footnoting of statistical methods in all tables and figures.

[3] Use appendices or supplements to house extended statistical details and scripts.

## Limitations:

This review was limited to one calendar year and focused on a single high-impact prosthodontic journal. Although extensive, the scoring still involved a degree of subjective interpretation. Furthermore, categorization of statistical appropriateness is inherently limited when studies underreport test assumptions or fail to specify methods explicitly.

## Conclusion:

This audit highlights the ongoing need for improved statistical literacy, transparency, and accountability in prosthodontic research. With less than half of studies exhibiting statistically rigorous methodologies, and nearly one-fifth showing clear misuse of statistics, the reliability of reported findings remains a significant concern. Hence, enhanced editorial standards, statistical training, and peer-review reform are critical steps to safeguard the integrity and credibility of evidence-based prosthodontics.

## Figures and Tables

**Figure 1:**
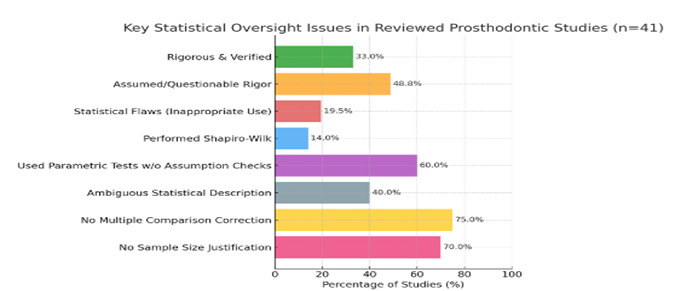
Key statistical oversight issues

**Table 1:** Categorization of studies based on statistical appropriateness

**Category**	**Definition**	**N**	**%**
Appropriate use	Statistical tests are fully justified, matched to data, assumptions checked	14	34.10%
Assumed appropriate use	Usage appears correct but lacks full justification or assumption testing	11	26.80%
Questionably appropriate	Somewhat reasonable use but lacks clarity, assumptions unchecked, or improper tailoring	9	22.00%
Inappropriate usage	Tests misapplied, mismatched with data, misleading or missing justification	5	12.20%
Serious statistical flaws	Critical issues such as false conclusions, misinterpretation, lack of any proper testing	2	4.90%
